# The Effects of Accelerated Temperature-Controlled Stability Systems on the Release Profile of Primary Bile Acid-Based Delivery Microcapsules

**DOI:** 10.3390/pharmaceutics13101667

**Published:** 2021-10-12

**Authors:** Armin Mooranian, Louise Carey, Corina Mihaela Ionescu, Daniel Walker, Melissa Jones, Susbin Raj Wagle, Bozica Kovacevic, Thomas Foster, Jacqueline Chester, Edan Johnston, Momir Mikov, Hani Al-Salami

**Affiliations:** 1 The Biotechnology and Drug Development Research Laboratory, Curtin Medical School & Curtin Health Innovation Research Institute, Curtin University, Perth 6102, Australia; A.Mooranian@curtin.edu.au (A.M.); louise.carey@student.curtin.edu.au (L.C.); c.ionescu@postgrad.curtin.edu.au (C.M.I.); danieljcswalker@gmail.com (D.W.); melissa.a.jones@postgrad.curtin.edu.au (M.J.); susbinraj.wagle@postgrad.curtin.edu.au (S.R.W.); bozica.kovacevic@postgrad.curtin.edu.au (B.K.); thomas.p.foster@student.curtin.edu.au (T.F.); j.chester@student.curtin.edu.au (J.C.); edan.johnston@student.curtin.edu.au (E.J.); 2 Queen Elizabeth II Medical Centre, Hearing Therapeutics, Ear Science Institute Australia, Perth 6009, Australia; 3 Department of Pharmacology, Toxicology and Clinical Pharmacology, Faculty of Medicine, University of Novi Sad, 21101 Novi Sad, Serbia; mikovmomir@gmail.com

**Keywords:** microencapsulation, diabetes mellitus, bile acids, Eudragit, chenodeoxycholic acid

## Abstract

Introduction: Bile acid-based drug encapsulation for oral delivery has been recently explored in our laboratory and has shown to be beneficial in terms of drug-targeted delivery and release profile, but stability at various temperatures has not previously been examined; hence, this is the aim of this study. Methods: Various types of bile acid-based microcapsules containing the drug metformin were produced and tested for accelerated temperature-controlled profiles, as well as morphology, elemental composition, drug content, resilience, floatability, wettability and release profiles at various pH values. Results: Accelerated temperature-controlled analysis showed negligible effects on morphology, size, or shape at very low temperatures (below 0 °C), while higher temperatures (above 25 °C) caused alterations. Drug contents, morphology and elemental composition remained similar, while wettability and the release profiles showed formulation-dependent effects. Discussion and Conclusion: Results suggest that bile acid-based microcapsules containing metformin are affected by temperature; hence, their shelf life is likely to be affected by storage temperature, all of which have a direct impact on drug release and stability profiles.

## 1. Introduction

Bile acid-based microcapsules have been shown to improve the release profile and better target the oral delivery of lipophilic drugs [1]; however, their application in hydrophilic drugs, such as the first line antidiabetic metformin, and their stability at various temperatures have not been examined. The stability of encapsulated compounds is significant in maintaining optimal release profiles; thus, it determines storage conditions and shelf life. As such, screening utilising accelerated stability testing, as well as formulation design and processing methods, must be considered [2]. If temperature variations cause alterations to bile acid-based microcapsules, then shelf life is likely to be affected, which would have an impact on both release and stability, an important consideration in the ongoing development of these microcapsules. 

Accelerated temperature-controlled stability systems are an important tool in quality control and design of formulations for microencapsulated compounds [3]. He et al. developed accelerated temperature control protocols yielding comparable kinetic parameters to traditional isothermal protocols with significant time and cost saving [4]. Similarly, a study by Zhao et al. used programmed heat and humidification protocols to investigate drug stability to yield comparable results to standard isothermal experimental protocols in a simpler and quicker manner [5]. This approach has been applied to microcapsules by Waterman et al. and Zolnik et al., in which accelerated testing and a modified Arrhenius equation were both used to predict stability and shelf life in reduced time frames [2,3]. These approaches allow us to perform a rapid review of the impacts of temperature on drug release and stability profiles, as well as the subsequent impact on shelf life and the implications of differing environmental and storage conditions. 

The impacts of alterations in environmental and storage temperature on drug stability is an important consideration. Yoshioka et al. demonstrated that shelf life can be estimated using accelerated testing at 40 °C as a function of activation energy when accounting for assay and temperature accuracy with a conservative activation energy estimation. However, even slight (1 °C) deviations in temperature control can impact probability predictions [6]. Nakamura et al. highlighted the complexity of predicting stability when drugs are exposed to differing thermal conditions, such as in transit and mobile medical applications, as well as exposure of drugs to extreme thermal stress once dispensed, and the need to take into consideration heat transfer in estimating stability in uncontrolled temperature conditions [7]. Kadam et al. demonstrated that two heterogenous nanoemulsions produced by bath sonification were thermodynamically unstable and lost stability on storage at room temperature, which impacted droplet size for one emulsion and showed an increase in flocculation in the other [8]. Both emulsions showed a decrease in pH at room temperature. Recognising the potential for exposure to greatly variable temperature conditions is key and can inform production and development methods.

Production methods, particularly the temperature of production, particle size, water content and formulation factors, can have an impact on drug stability and release. A study by Sakurai et al. demonstrated that particle size, water content and production temperature all impacted stability and drug release [9]. Preparations with a lower water content produced by hot melt extrusion were more stable than those with a higher water content produced by spray drying, while smaller particle sizes resulted in reduced solubility and wetting properties, which highlighted the need to control particle size. Beirowski et al. investigated the role of excipients as lyoprotectants along with steric stabilisers in freeze-drying cycles of varying intensity, showing that primary cycles could be greatly shortened and highlighting the importance of formulation design in the stability of nanosuspensions [10]. Chen et al. similarly highlighted the role that formulation factors, in addition to processing parameters, particularly temperature, play in altering the release profile, with great differences between hot melt extruded formulations prepared at 190 °C and those prepared at 205 °C, from the four processing temperatures assessed [11]. Broadly, these studies highlighted the need for consideration of processing methods and, importantly, the formulation design in developing stable preparations with optimal release profiles; hence, consideration was taken in determining the formulations used in this study. 

The ratio and interaction of excipients with drugs, both physically and chemically, are an important consideration in drug development. Excipients can impact drug stability, influence degradation and also result in potentially toxic degradation products [12]. The ratio of drug to excipient was explored by Waterman et al., who showed that, when drug and excipient particles are compatible, the excipient’s impact on the degradation rate is independent from the drug concentration. However, when drug particle concentrations exceed excipient affinity concentrations, a power law relationship emerges following the surface-to-volume ratio of the amount of the drug present [13]. The encapsulation of metformin with alginate showed that the ratio of drug to excipient impacted the loading of metformin along with the production efficiency and release profiles [14]. Metformin has not yet been extensively studied in combination with a range of excipients, although it has been shown to interact with PVP, mannitol and magnesium stearate, resulting in impacts on chemical stability, as well as accelerated degradation [15]. Possible negative impacts, along with production efficiency, when utilising different excipients, is central to the effective development of stable and safe drug delivery systems. Hence, impacts of excipients and concentrations of each must be accounted for when designing systems and formulations for drug release assessments. 

Polymers are commonly used excipients in formulations, often serving as the coating material offering an enhanced stability [16]. Membrane materials, including polymers, should be chosen based upon their properties, including the influence that varying conditions have on their structure and function. Such conditions include pH and temperature-changing stimuli [17]. Eudragit polymers are marketed as being pH sensitive, due to their varying solubilities in various pH values. Therefore, when encapsulated, Eudragit polymers would impact drug release concentrations in specific pH values [18]. 

To further investigate the incorporation of chenodeoxycholic acid (CDCA) and impacts of temperature on drug stability, various formulations of polymer-CDCA microcapsules containing metformin were developed. These formulations were tested for accelerated temperature-controlled profiles as well as morphology, elemental composiftion, drug content, resilience, floatability, wettability and release profiles at various pH values. Formulations both with and without CDCA were investigated to elucidate the impact of drug-excipient interaction and subsequent impact on stability and release profiles. 

## 2. Materials and Methods

### 2.1. Materials

Metformin (M, 98%), sodium alginate (SA, 99%), chenodeoxycholic acid (CDCA, 99%) and Poloxamer 407 were purchased from Sigma Chemical Co (St. Louis, MO, USA). Eudragit^®^ RS30D, RL30D and NM30D polymers were supplied by Evonik Industries (Essen, Germany). Calcium chloride dihydrate (CaCl_2_ 2H_2_O 98%) was obtained from Scharlab S.L (Barcelona, Spain). All reagents and solvents were HPLC grade, used without further purification or modification and were supplied by Merck (Bayswater, VIC, Australia).

### 2.2. Drug Preparations

Stock suspensions of M (0.4 mg/mL) and CDCA (0.3 mg/mL) were each prepared by placing 100 mL of ultrapure water on a magnetic stirrer and adding the % by weight of reagents. Suspensions were left to mix thoroughly at room temperature for 4 h, stored in the refrigerator and used within 48 h of preparation. A stock solution of CaCl_2_ (2%) was prepared by adding CaCl_2_ powder to HPLC water and mixed thoroughly as above.

### 2.3. Preparation of Microcapsules

Microcapsules were produced using a Büchi microencapsulating system (Büchi Labortechnik, Flawil, Switzerland), according to previously established methods [19,20,21], for all 6 formulations,. Formulations 1-3 (F1–F3) were made of SA, M, one type of Eudragit (NM30D, RL30D, RS30D) and Poloxamer 407 in concentrations (% *w*/*v*) of 1.6, 0.4, 2 and 4 respectively, while formulations 4–6 (F4–F6) were made of the same constituents with the addition of 0.3% CDCA. Microcapsules were freshly prepared, stored in the refrigerator and used within 48 h. This method utilises our established vibrational jet flow technique for microencapsulation of polymer solutions. Parameters were set at a flow rate of 2 mL/min, with a frequency of 1000–1500 Hz under a constant pressure of 300 mbar. Such parameters were selected for their production of idealistic microcapsules, according to previously established methods. Microcapsules were left in the CaCl_2_ gelation bath for 5 min and then collected. Each formulation was prepared and tested separately, with three independent batches per formulation. All microcapsules were prepared and treated in the same way, irrespective of formulation [22].

### 2.4. Characterization of Loaded Microcapsules

#### 2.4.1. Microcapsule Morphology and Surface Analysis

The structure and morphological characteristics of capsules were examined using an Olympus IX-51 inverted microscope (Olympus Company, Tokyo, Japan), using brightfield light [23].

Microcapsule surface morphology was examined using MIRA3 FibSEM (Tescan, Brno, Czech Republic) with a 2.5 nm calibrated resolution at 3 kV. Prior to scanning, the samples were mounted on a glass stub and coated under vacuum in an argon atmosphere with platinum (5 nm), as per our established methods [24,25,26].

#### 2.4.2. Microcapsule Resistance and Buoyancy 

The mechanical stability of the microcapsules was tested using a Boeco Multishaker PSU 20 (Hamburg, Germany) over a period of 72 h, according to established laboratory methods [27]. At 12-hour intervals, the number of damaged or fractured capsules was recorded. Briefly, 100 capsules were incubated in 5 mL of phosphate buffer at 37 °C and agitated at 150 rpm. The mechanical resistance was measured as a percentage of the number of intact remaining capsules over the initial number of capsules [28].

The buoyancy of microcapsules was tested utilising a USP dissolution apparatus 24 (Type II). Two grams of microcapsules were placed in 200 mL of simulated intestinal fluid (phosphate buffer, pH 7.8). The solution was stirred at 100 rpm by rotating paddles at 37 °C for 24 h and the buoyancy was expressed as a percentage of capsules floating over initial number of capsules [29,30].

#### 2.4.3. Swelling Studies

Swelling studies were carried out over 4 hours, with 100 capsules placed in 5 mL of buffer at two temperatures (25 °C and 37 °C) and two pH values (3 and 7.8). At hourly intervals, the capsules were removed, blotted dry with filter paper and weighed on an electronic balance. The swelling index was calculated as a percentage of final weight over initial weight [22,31].

#### 2.4.4. Accelerated Stability Testing

Set amounts of freshly prepared microcapsules placed in sterile petri dishes and stored in a stability chamber (Angelantoni Environmental and Climatic Test Chamber, Italy) for 7 days at the temperatures of −20 °C, 5 °C, 25 °C and 37 °C and relative humidity of 35%, as per established protocols [32,33]. At the conclusion of the study the microcapsules were analysed for visual changes in colour, morphology and appearance [34].

#### 2.4.5. Drug Release Studies and High-Performance Liquid Chromatography (HPLC) Instrumentation and Chromatographic Conditions

To determine the release profile of metformin from the microcapsules, one gram of capsules was weighed and placed in 200 mL of buffer at 4 different pH values (1.5, 3.0, 6.0 and 7.8) and agitated at 200 rpm for 6 h. At predetermined time points, 2 mL aliquots of the release medium were withdrawn and measured for absorbance using a LAMBDA 25 UV-Vis spectrophotometer (Perkin Elmer, Waltham, MA, USA), as per well-established methods [32,35]. Metformin concentrations were determined at 233 nm against the calibration curve, with the buffer as blank. Sink conditions were maintained throughout via the use of a closed-loop system, with average values calculated with all analyses carried out in triplicate [34].

The drug content was calculated using previously established HPLC methods using a reversed phase Shimadzu HPLC (Kyoto, Japan) equipped with an SPD-20A UV detector [36,37]. Isocratic conditions were maintained and an Alltima CN (250 mm × 4.6 mm × 5 μ) was used with a retention time of 6.9 min, injection volume of 20 µL and flow rate of 1 mL/min. Known concentrations of metformin were used to construct a calibration curve. One gram of microcapsules was dissolved in 2 mL of mobile phase (20 mM ammonium formate buffer, pH 3.5 and acetonitrile, 44:55, *v*/*v*) and centrifuged at 1500 rpm for 15 minutes, after which the supernatant was removed for the HPLC analysis. 

#### 2.4.6. Liquid Chromatography Mass Spectrometry (LC-MS) Instrumentation and Chromatographic Conditions

The quantification of bile acid in the microcapsules containing CDCA (F4–6) was undertaken utilising a LC-MS analysis on a Shimadzu LCMS 2020 system (Kyoto, Japan) with separation by a Phenomenex C-18 column (5 µm, 100 mm × 2 mm), according to well validated methods [38]. As in the HPLC analysis, 1 g of microcapsules was dissolved in 2 mL of mobile phase (methanol and water, 65:35 *v*/*v*, at pH 2.9) with a retention time of 2.9 mins, injection volume of 50 µL and flow rate of 0.25 mL/min [38,39].

### 2.5. Statistical Analysis 

Graph Pad Prism version 8.2 (Graphpad, Inc., San Diego, CA, USA) was used to create a graph and the results are presented as mean ± SD. The statistical measurements were carried out using a parametric/non-parametric analysis or using a one-way ANOVA and a Tukey’s post hoc test, at the appropriate level of significance (*p* > 0.05).

## 3. Results and Discussion

### 3.1. Scanning Electron Microscopy (SEM) and Energy Dispersive X-ray (EDXR) Spectroscopy

**Figure 1:**
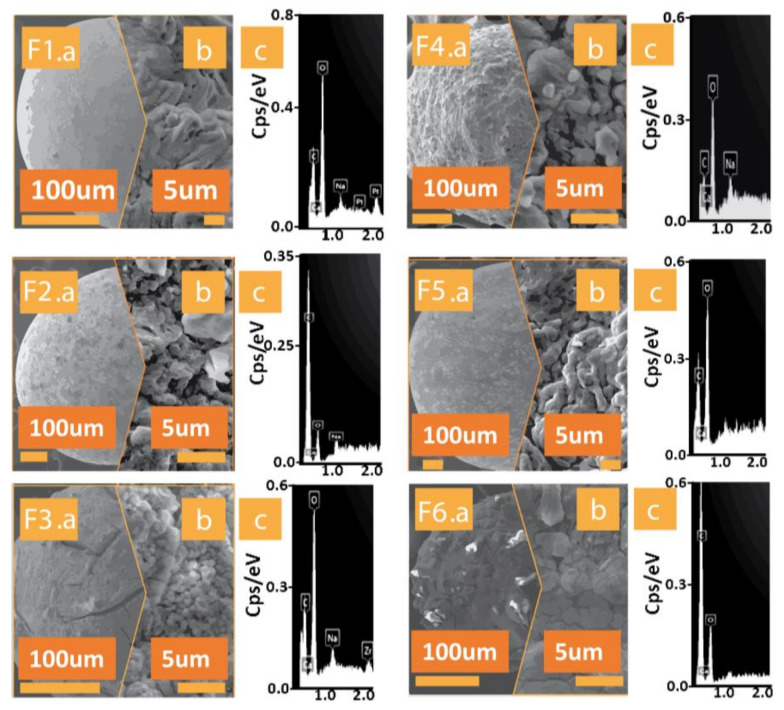
Scanning electron micrographs and surface morphology of formulations from F1 to F6 and corresponding surface morphology (labelled (**a**) for scale of 100 µm and (**b**) for scale of 5 µm)., and energy dispersive x-ray from F1 to F6 (labelled as (**c**) for each) showing surface elemental composition.

The microcapsules were analysed for morphology (Figure 1a for formulations from F1 to F6) and surface topography (Figure 1b for formulations from F1 to F6) via SEM with randomly selected samples from each batch. This revealed a similar spherical shape across the capsules, with similar size between control and test capsule pairs. Crystallisation can be seen on the capsule surfaces (Figure 1b). The similarity between the test formulations and controls indicates that the added excipients do not negatively impact microcapsule size. As discussed in the introduction, particle size impacts solubility and wetting properties [9]. Hence, size is an important consideration in this study.

EDXR allows for the elemental composition of the surface of the capsules to be identified, with coating materials neutralized by the instrument. Figure 1 (Figure 1c for formulations from F1 to F6) shows the surface elemental composition of each of the formulations. Predominant elements are oxygen, calcium, sodium and chloride, which is expected for microcapsules produced by ionic gelation, given the CaCl_2_ gelation bath and incorporation of sodium alginate. All formulations (control and test) showed similar surface elemental composition. 

### 3.2. Drug and CDCA Contents of Microcapsules

**Figure 2:**
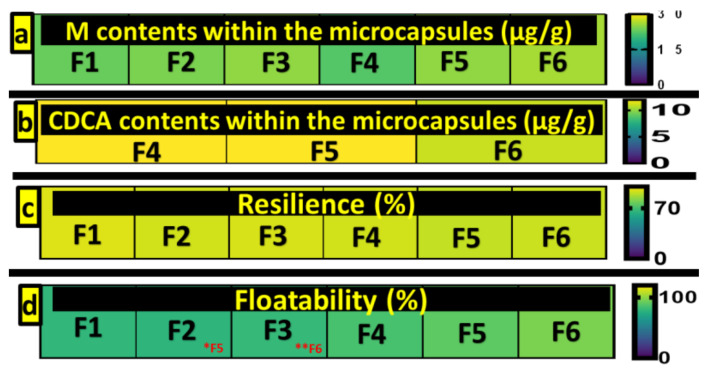
Metformin contents of microcapsules (**a**), CDCA contents of microcapsules (**b**), mechanical resistance of microcapsules (**c**) and buoyancy of microcapsules (**d**).

Figure 2 (1 and 2) shows the drug contents of the microcapsules from the HPLC analysis. There was no significant difference between the formulations in amount of metformin encapsulated (*p* > 0.05), with content across formulations uniformly high illustrating the efficiency of the encapsulation method. Additionally, there was no significant difference in CDCA loading between formulations incorporating it, *p* > 0.05 (F4–6). This shows that CDCA has no impact on metformin encapsulation or the encapsulation process which aligns with our previously published work incorporating bile acids into microcapsules [31,33]. 

### 3.3. Mechanical Strength and Buoyancy 

The mechanical strength of microcapsules is outlined in Figure 2. There was no significant difference in resilience among formulations, *p* > 0.05, with all formulations showing high resistance to mechanical stress. This indicates that capsules are resilient enough to withstand the mechanical stresses of peristaltic contraction in the gastrointestinal tract. It also demonstrates that the addition of CDCA does not negatively impact the resilience of the microcapsules. 

The buoyancy tests revealed significant changes between F6 and F3, with the same Eudragit, RS30D, contained, but F6 also having CDCA, *p* < 0.05. Similarly, in the case of F2 and F5, which also had equal Eudragit (RL30D), F5, containing CDCA, had significantly more floatability than F2, which did not contain CDCA. The results are indicative that the addition of CDCA does not negatively impact the buoyancy of capsules, but improves it instead, in the case of F5 and F6 (Figure 2 (4)). The buoyancy values indicate the microcapsules’ ability to float and remain in the gastrointestinal tract. High floatability may reduce hinderance by gut mucosa and enhance retention time, allowing for the sustained release of the encapsulated drug [29,40]. All capsules showed high buoyancy under the simulated gastric fluid conditions, allowing them to swell sufficiently to release metformin in a steady manner. High floatability, combined with the high mechanical strength and resistance to shear stress, indicates capsules are resistant to abrupt rupture resulting in irregular drug release and have desirable characteristics for controlled release.

### 3.4. Accelerated Stability Testing

**Figure 3:**
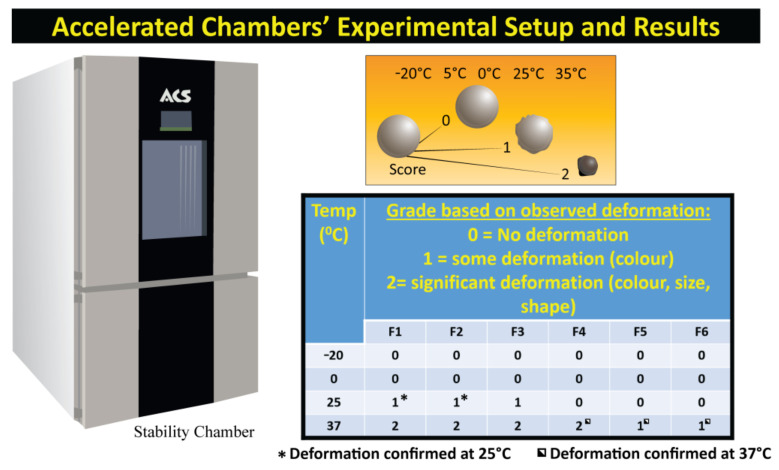
Accelerated stability testing setup, deformation key and results.

Figure 3 outlines microcapsules deformation following stability testing. Accelerated stability testing allows us to analyse environmentally induced changes to the microcapsules in terms of size, shape and colour which may affect drug stability and have implications for storage conditions [41]. At lower temperatures (−20 °C and 0 °C), all formulations retained the original characteristics, showing no changes in colour, morphology, or size. At 25 °C, the formulations not including CDCA (F1–3) began to show lower grade deformation with changes in colour, while those containing CDCA retained original size and characteristics. At 37 °C, all formulations showed deformation, with all capsules not containing bile acid showing deformation with changes in colour, shape and size. F4 also showed deformation. F5 and F6 showed some deformation with changes in colour recorded at 37 °C. Colour changes are due to the oxidisation of the alginate, while size and shape changes could be due to the loss of water and subsequent dehydration, which is in line with our previous work with alginate-based capsules [21,32]. Temperature-induced changes have a great impact due to the implications for the preparation and storage conditions of capsules before use, as well as ensuring precise delivery of drugs [42,43]. These results indicate that the incorporation of CDCA improves the stability of microcapsules depending on formulation. 

Excipients have been shown to impact the stability of pharmaceutical formulations which can result in reduced potency and performance of drugs. Accelerated temperature testing is a way to screen for compatibility of drugs and excipients to allow for more efficient formulation development [12]. Previous studies undertaken by our lab incorporating CDCA into sodium alginate-based capsules have shown similar grade deformation due to dehydration and oxidation, with no impact on drug structure or content compared to freshly made capsules [21,28,34]. While drug content, thermal and chemical stability analyses post stability testing were not undertaken in this study, it is expected that, as per our previous work, capsule stability, as well as drug content and release, would not be impacted by minor deformations.

### 3.5. Swelling Studies

**Figure 4:**
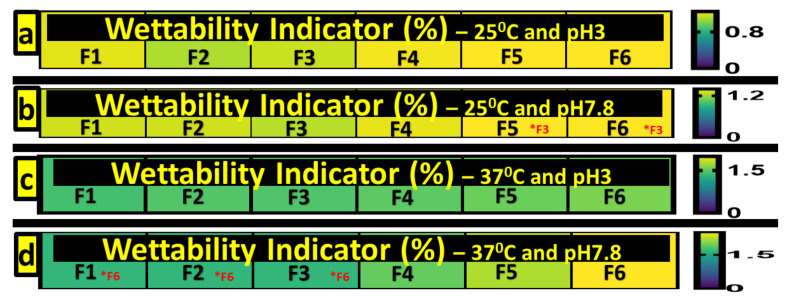
Swelling studies at 25 °C and pH 3 (**a**), 25 °C and pH 7.8 (**b**), 37 °C and pH 3 (**c**) and 37 °C and pH 7.8 (**d**).

Swelling indexes indicate wettability, that is, the water penetration and subsequent expansion of capsules. This is dependent on characteristics of the capsule membrane, such as polarity and how porous the surface is, impacting the amount of water a capsule can absorb, as well as conditions including temperature and pH. Swelling is an important indicator, as it can influence the drug release profiles of capsules, with higher swelling indicating a corresponding higher release. Figure 4 (2 and 4) shows that pH and temperature impact the wettability of the microcapsules in a formulation-dependent manner. 

**Figure 5:**
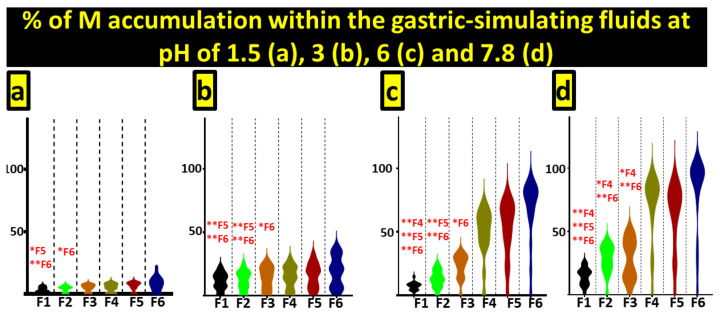
Metformin release in simulated gastric fluids at pH 1.5 (**a**), pH 3 (**b**), pH 6 (**c**) and pH 7.8 (**d**).

Swelling at pH 3, for both 25 °C and 37 °C, showed no significant differences between formulations (*p* > 0.05), showing that the incorporation of CDCA did not impact wettability at undesirable pH levels. However, at pH 7.8, differences emerged at both temperatures tested, with body temperature (37 °C) yielding the most differences. This is due to increased water bonding, water penetration and subsequent expansion [44]. F5 and F6 showed differences, with F6 yielding the most difference, with statistically significant values compared with F1, F2 and F3 in wettability at 37 °C and pH 7.8. In terms of F3 and F6, both containing Eudragit RS30D, F6, with the addition of CDCA, had a statistically significantly higher wettability than F3 at pH 7.8 and both temperatures, 25 °C and 37 °C (*p* < 0.05). This shows the impact that the addition of CDCA has on the formulations’ wettability results. The results at pH 7.8 and 37 °C are representative of the distal small intestine, where metformin is primarily absorbed. The incorporation of CDCA increased the swelling index, thus increasing drug release at specific conditions amenable to an improved delivery of metformin. This correlates with subsequent drug release data with higher swelling aligning with higher drug release at the same conditions (Figure 5).

### 3.6. Drug Release Studies

Figure 5 outlines the release profile of metformin from microcapsules at the different pH values reflecting gut segments. These simulated conditions were selected based on a large body of previous work exploring the release of drugs at different gut segments [31,32,33,45]. The release of metformin varied according to pH, with levels reflecting the upper GIT (pH 1.5 and 3) showing minimal release, while, at higher pH simulating the small intestine (pH 6 and 7.8), the release of metformin increased, with the highest release occurring at a pH of 7.8. 

In oral drug delivery, the influence of pH is key in drug release, particularly given the changes in pH throughout the gastrointestinal tract and the need for microcapsules to survive the highly acidic environment of the stomach. Eudragit incorporation in drug delivery systems has long been established in improving controlled drug release by increasing resistance to acidic pH levels. Improvements have been noted when incorporating the co-polymer compared to the commercially available product, resulting in release further down the gastrointestinal tract at optimal sites for absorption, in a formulation-dependent manner [46,47]. More recent work has shown Eudragit optimises drug release, allowing for targeted and controlled release dependent on pH, again depending on formulation [48,49,50]. The incorporation of co-polymers and bile acids has been explored by our lab with positive effects on stability and release for orally delivered drugs [51]. Metformin is primarily absorbed at the small intestine and has low oral bioavailability and low membrane permeability due to its pharmacokinetics at physiological pH [52,53]. Absorption occurs via passive diffusion as well as saturable active transport via organic cation transporters with a high variability in absorption depending on glycaemic state and biological factors [54]. While metformin is extremely effective at a population level, there is also a high individual variability in response to metformin, which is associated with genetic variation in biological responses and is heritable [55]. Up to 30% of patients report gastrointestinal side effects which can lead to discontinuation of treatment in a large number, due to the high doses required to overcome its limited bioavailability and flip-flop pharmacokinetics [56]. Modified release formulations have been developed to try and reduce these impacts but have shown no reduction in side effects [57,58]. Microencapsulation provides a delivery method that can work to overcome pharmacokinetic limitations and improve therapeutic effects [59]. Formulations containing CDCA showed more metformin release at pH 7.8 than formulations without, indicating that the inclusion of CDCA improved the release profile. 

At pH 7.8, formulations without CDCA showed irregular and incomplete release patterns, while the addition of CDCA improved the release pattern. F4–6 all showed more controlled release at the target pH, along with complete release of encapsulated metformin. Furthermore, in terms of F1 and F4, both containing Eudragit NM30D, the addition of CDCA showed a higher metformin release profile, which was statistically significant at both pH 6.0 (*p* < 0.05) and pH 7.8 (*p* < 0.05). The benefit of the inclusion of CDCA can also be seen from F2 and F5, both with the polymer Eudragit RL30D, in which F5 showed a better release profile, including significant results at pH 3.0 (*p* < 0.05) and pH 6.0 (*p* < 0.05). The comparison of the results from F3 and F6 also demonstrates a similar positive benefit to the inclusion of CDCA in the formulations, with both F3 and F6 containing Eudragit RS30D and showing a significantly improved metformin release for F6 at pH 3.0, pH 6.0 and pH 7.8 (all *p* < 0.05). 

This release profile of CDCA improving the results in F4, F5 and F6 aligns with the increased swelling of these formulations under the same conditions, allowing for release of the encapsulated drug. As metformin is absorbed primarily in the small intestine, the targeting of release at pH 7.8 works to maximise absorption and can work to overcome individual variability and improve bioavailability, while also reducing side effects, as lower dosages are required when drug delivery is targeted and controlled.

## 4. Conclusion

The addition of CDCA to polymer-based microcapsules containing metformin resulted in improved drug delivery and release at the simulated target pH of the small intestine without compromising capsule morphology, size, or physical characteristics. Accelerated stability testing showed that the incorporation of CDCA improved stability in a formulation-dependent manner, with two formulations incorporating CDCA being resistant to degradation at temperatures below 37 °C, compared to the controls without CDCA, that showed deformation from temperatures of 20 °C. This has implications not only for storage and transport, but also for drug stability and release. Further research into release profiles of capsules subjected to high temperature stability testing is required to establish the potential requirements for capsules to have ascertained the optimization of specific storage conditions in order to remain stable and maintain improved drug release, or to further optimise formulations to improve temperature stability.

## Data Availability

The data presented in this study are available on request from the corresponding author. The data are not publicly available due to author property agreements.
